# A unique presentation of echo phenomena in a patient with catatonia: a case report and literature review

**DOI:** 10.1186/s12888-023-04821-w

**Published:** 2023-05-24

**Authors:** Dallas Hamlin, Yassir Mahgoub

**Affiliations:** Department of Psychiatry and Behavioral Health, Penn State Health Milton S Hershey Medical Center, Hershey, PA USA

**Keywords:** Catatonia, Case Report, Auditory Hallucination, Echolalia

## Abstract

**Background:**

Catatonia is a complex syndrome notable for a highly variable presentation. Standardized exam and criteria can enumerate possible presentations, but recognition of novel catatonic phenomenon may facilitate better understanding of catatonia’s core features.

**Case presentation:**

A 61 year-old divorced pensioner with history of schizoaffective disorder was hospitalized for psychosis in the setting of medication noncompliance. While hospitalized, she developed multiple classic catatonia signs such as staring and grimacing, as well as a bizarre echo phenomenon while reading text that improved alongside other catatonic symptoms with treatment.

**Conclusion:**

Echo phenomenon are a component of catatonia often recognized when presenting as echopraxia or echolalia, but other echo phenomenon are well established in the literature. Recognition or novel catatonic symptoms like this can lead to improved recognition and treatment of catatonia.

## Background

Catatonia is a complex psychomotor syndrome seen in multiple psychiatric illnesses, with a prevalence as high as 10–25% among inpatient populations [1, 2]. Catatonia can have many signs and symptoms, with a contemporary review noting as many as 40 [3]. The DSM-5-TR recognizes echolalia and echopraxia as potential echo phenomenon and describes them as “mimicking another’s speech” and “mimicking another’s movements,” respectively [4]. A broadened conceptualization of the echo phenomenon may facilitate detection and treatment of subtle or nonclassical presentations of catatonia. Herein, we present a case of a unique echo-phenomenon demonstrated by a patient admitted to our inpatient service to illustrate this point.

## Case report

Ms. G was a 61 year-old divorced woman with a remote medical history of renal transplant maintained on azathioprine 125 mg daily and prednisone 5 mg daily and a psychiatric history of schizoaffective disorder maintained on ziprasidone 20 mg twice daily. She was brought to the hospital due to a subacute history of progressive inability to care for self, poor sleep, and psychomotor agitation in the setting of a several month history of derogatory auditory hallucinations, paranoia towards family, and the delusional belief that the hallucinations were due to a transmitter placed in her head. She was brought to the medical hospital by family, who denied any other family history of psychiatric illness. They reported that the patient was independent with activities of daily living and otherwise with good functioning when compliant with medication. She had had multiple prior psychiatric hospitalizations due to psychosis in the setting of medication non-compliance. Prior medication trials included olanzapine and risperidone, but these were discontinued for oversedation and tremors, respectively. Medical work-up revealed a urinary tract infection treated with cefuroxime, but it was otherwise unremarkable with respect to infectious workup including comprehensive metabolic panel, complete blood counts, and thyroid stimulating hormone. The patient was irritable on initial interview and demonstrated multiple signs of catatonia over 24 h of observation such as grimacing, negativism, manneristic and high-pitched speech, staring, and echopraxia, leading to a cumulative Bush-Francis Catatonia Rating Scale (BFCRS) score of 12. The patient’s home medication of ziprasidone was resumed at 20 mg twice daily.

The patient’s symptoms fluctuated with alternating periods of stupor, psychomotor agitation, grimacing, and manneristic speech, but the length and frequency of these episodes decreased as ziprasidone was up-titrated. On day 3 of hospitalization, the patient was sufficiently lucid to discuss her symptoms with the treatment team. She endorsed significant paranoia towards family, continued hallucinations, and the desire to read a book to distract herself. The next day, the patient reported a new hallucinatory experience distinct from her presenting auditory hallucinations. She reported that whenever she read the text of a book, she would hear a different voice from her typical auditory hallucinations yelling the text of the book at her. This would stop immediately when she closed the book, and it would begin again once she re-opened a book. This improved as ziprasidone was titrated to a final dose of 40 mg in the morning and 60 mg in the evening, and her derogatory auditory hallucinations and delusional beliefs also abated after 3 weeks of treatment. During this time, the patient’s periods of irritability resolved, and they were generally pleasant and engaged in the inpatient milieu. Supportive psychotherapy techniques were utilized throughout hospitalization, and the patient reported benefit from this and pharmacological interventions at the conclusion of her hospitalization. The patient was discharged to outpatient follow-up without residual psychotic or catatonic symptoms and fair insight.

## Discussion

Echolalia and Echopraxia are commonly recognized signs of catatonia noted in resources such as the DSM-5-TR [4] or the BFCRS [5] They are commonly referred to jointly as echo phenomenon. They are so named in that they are automatic and stimulus-bound in nature, providing a vocal or motoric copy of an environmental cue.

This straight-forward definition of echo phenomenon was noted in disorders as diverse as catatonia, schizophrenia, epilepsy, affective disorders, and neurodevelopmental disorders. Early analysts such as Stengel (1947) related this phenomenon to normal motor and speech development, and further conceptualized seemingly purposeless repetition of complex behaviors and speech topics as other forms of echo phenomenon [6]. Building upon this conceptualization, one contemporary review describes a multiplicity of echo phenomenon [7]. Among the included examples were echoplasia (repetitive mental or physical act of tracing the contours of a certain human or object, in the air, or on a given surface), echograpia (written repetition of usually verbal stimuli), and echolalioplasia (repetitive use of motor actions like sign language to echo verbal stimuli) [8–10]. These phenomena differ from echopraxia and echolalia in that the repetitious act is not in the same modality as the stimulus. This also demonstrates that echo phenomenon can include complex behaviors such as writing.

In addition to complex behaviors, there are further nuances of catatonic speech relevant to echo phenomenon. While describing catatonic speech disorders, Ungvari et al. [11] discuss the phenomenon of “Mental echolalia” (echoing only in mind) and “hallucinatory echolalia” (repeating one’s own hallucinations) as related phenomenon. This demonstrates that the patient’s echo response may be a mental phenomenon instead of an observable behavior, and the stimulus itself may be hallucinatory as in the case of hallucinatory echolalia. The latter bears resemblance to the patient described in this case report. However, the hallucinatory echolalia phenomenon represents a verbal output to what the patient experiences as auditory stimuli, and the patient in this case experienced auditory hallucinations as an output from written stimuli. Multiple reviews include further reports that the repeated response may even be temporally decoupled from the stimulus, as is the case in delayed echolalia [7, 11]. As with echoplasia, echographia, and echolalioplasia, these phenomena are stimulus bound and are recapitulatory in nature, though they are not necessarily in the same modality of the stimulus as is the case in echopraxia or echolalia.

This distinction has important implications for the detection and management of catatonia, as in the case of this patient. The patient’s own echo phenomenon suggested the continued presence of catatonic symptoms, which could be misinterpreted as signs of schizophrenia. This patient presented with manic symptoms in the setting of several months of psychotic symptoms, consistent with her historical diagnosis of schizoaffective disorder with catatonic features. This patient’s case is also notable for a varied and rapid presentation of catatonia that improved without the first-line therapy of benzodiazepines. However, catatonia is known to have a fluctuating natural history, and precedent exists for catatonia resolving with treatment of underlying affective illnesses [12]. Some resources, including the Maudsley prescribing guidelines, recommend trialing atypical antipsychotics if there is not a concern for neuroleptic malignant syndrome [13]. Despite this patient’s presentation with multiple catatonic signs and successful response to antipsychotics, it must be noted that catatonia is both under-recognized and under-treated [14], so subtle signs and symptoms of catatonia must be recognized to hasten diagnosis and treatment. The patient fortuitously responded well to initial treatment selection, though it is known that catatonic symptoms can worsen with antipsychotics.

In conclusion, if the premise of a diverse range of echo phenomenon is accepted, it stands to reason that there are as of yet undescribed echo phenomenon that can be observed in the diverse range of disorders that present with echo phenomenon such as catatonia, schizophrenia, autism spectrum disorder, and Tourette’s Syndrome [7]. These phenomenologically distinct signs and symptoms may evade clinical notice if diagnostic frameworks are limited to isolated presentations of phenomenon that can present in a multiplicity of unique ways. Frameworks for catatonia that can flexibly accommodate novel presentations of the syndrome may be of clinical utility in the future. Though the etiology of these phenomenon remains unclear, this case highlights the importance of careful dissection of the phenomenological experience of the patient, for catatonic symptoms can take varied and novel forms.

## Data Availability

Not Applicable.
